# Cohort profile: mental health following extreme trauma in a northern Ugandan cohort of *W*ar-*A*ffected *Y*outh *S*tudy (The WAYS Study)

**DOI:** 10.1186/2193-1801-2-300

**Published:** 2013-07-03

**Authors:** Kennedy Amone-P’Olak, Peter B Jones, Rosemary Abbott, Richard Meiser-Stedman, Emilio Ovuga, Tim J Croudace

**Affiliations:** Department of Psychology, Gulu University, P O Box 166, Gulu, UGANDA; Department of Psychiatry, University of Cambridge, Cambridge, UK; Department of Psychiatry and Mental Health, Gulu University, P O Box 166, Gulu, UGANDA; MRC Cognition and Brain Sciences Unit, 15 Chaucer Road, CB2 7EF Cambridge, UK; Department of Health Sciences, Seebohm Rowntree Building, University of York, Heslington, York YO10 5DD UK

## Abstract

War experiences are associated with the risk of long-term mental health problems. The War-affected Youths (WAYS) Study comprises a cohort of 539 youths (61% female) aged between 18 to 25 (at baseline) randomly sampled from the population of war-affected youths in northern Uganda. The study aims to chart the trajectory of long-term mental health consequences of war and the roles of individual, family, and community contextual risk and protective factors in influencing the course of mental health using Social Ecology Model, thus, addressing both the individual and its social ecology. Knowledge of postwar contexts may inform policy and guide interventions on postwar psychosocial adjustment and reintegration in conflict-prone Great Lakes region of Africa (Rwanda, Burundi, DR Congo, Uganda, Central African Republic, and South Sudan). Two waves of data collection have been conducted and more data collection is planned. At baseline, information on demographic characteristics, pre-war experiences, psychosocial outcomes, coping, stigma/discrimination, family and community acceptance and relationship, family functioning, and post-war experiences were obtained. At follow-up, information on general health, gender-based violence, PTSD, social skills, trauma memory quality, rumination, self-esteem, and psychosocial outcomes were collected. Approval to access the data can be obtained on application to the Principal Investigator upon submission of a research proposal with ethical approval from the applicant's institution. This research is funded by Wellcome Trust and Gulu University.

## Introduction

**Figure 1:**
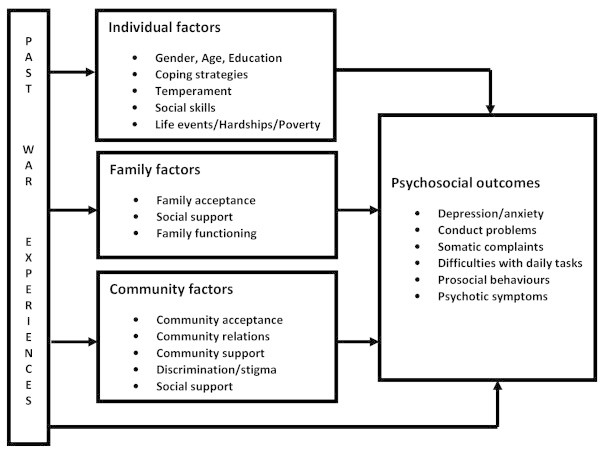
**Mediation and moderation by contextual factors.**

The aftermath of war includes long-term effects on mental health. Some studies report a dose–response relationship between mental health and severity, number or duration of exposure to war events (Fayyad et al. 2004). Others suggest differential pathways between particular war experiences and specific mental health outcomes while other studies report a gender-specific course (Layne et al. 2010; Pat-Horenczyk et al. 2009; Shaw 2003). Such a range of findings may be due to different post-war individual, family and community contexts including certain war events that vary by sex, social skills deficits in building social networks after war, poor family functioning, and experiences of discrimination in the communities to which those affected by war return (Betancourt et al. 2010). In this research project, we aim to assess the long-term mental health of former child soldiers (now youths) in northern Uganda aim to examine the extent to which individual, family and community risks or protective factors influence the long-term mental health outcomes in veteran child soldiers to gain insight into the mechanisms between war experiences and mental health outcomes. In general, we theorise that post-war mental health outcomes are likely to be influenced by the interactions between individual, family and community contextual factors (Figure 1).

### The use of child soldiers in Uganda

**Figure 2:**
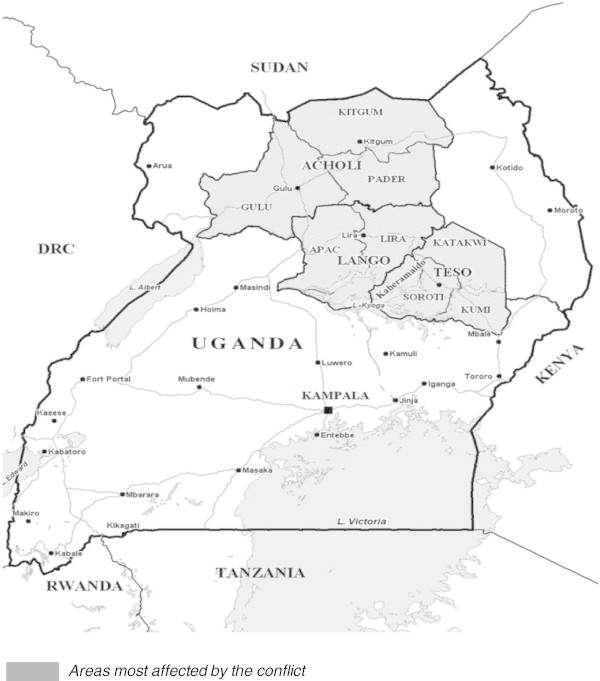
**Map of Uganda.**

A brief outline of the history of child soldiering in Uganda is necessary to put the study in context. Children were used in two recent violent wars that occurred in Uganda since 1980. The first was the National Resistance Army (NRA) guerrilla war from 1981 to 1986 where an estimated 3,000 children were used as soldiers (Schubert 2006). The second was the Lord’s Resistance Army (LRA) war from 1986 to 2006, where an estimated 30,000 children mainly from the Acholi ethnic group were forcefully abducted and recruited into the ranks of LRA (UNICEF, 1998; Women's Commission report, 2001; Kibanja et al. 2012). Child fighters constituted about 30 per cent of all NRA fighters and about 85 per cent of LRA fighters (Schubert 2006; UNICEF 1998). This study will focus on the youths affected by the LRA war where the abducted children were used as combat troops, spies, porters, “wives”, and human shields (Ehrenreich, 1997; Amone-P’Olak 2004; Amone-P’Olak 2005; Amone-P’Olak 2006; Amone-P’Olak et al. 2007). In captivity, the abductees lived in constant terror of attacks from government soldiers, threat of death, diseases, extreme deprivations, and, above all, sexual abuse and torture (Amone-P’Olak 2009). The children and adolescents were forced to kill, mutilate, torture, raid, and burn villages. In a strategy known as “burning the bridge” the abducted children were forced to loot and commit other atrocities against each other and against their own communities (Women's Commission report 2001; Kibanja et al. 2012). This strategy was aimed at deterring abductees from escaping and severing the bond between the abductees and their communities (Amone-P’Olak 2004). At the height of the war, more than 90% of the population in the war-affected districts of northern Uganda was displaced as indicated in Figure 2 (UNICEF 1998). Despite the “burning the bridge” strategy, many of the abducted children (now youths) escaped from rebel captivity and are currently living in communities in northern Uganda (Amone-P’Olak 2004).

### How did the study come about?

The Wellcome Trust started a research and training initiative in East Africa in 2009 to develop the expertise and international collaborative potential of indigenous scientists and researchers in health sciences research. Through this project, a new longitudinal research project was started at Gulu University, located in northern Uganda, in collaboration with University of Cambridge in the United Kingdom.

The War-Affected YouthS (WAYS) survey was conceived in order to study the psychosocial outcomes of exposure to war without the limitations of previous research into the sequelae of conflicts in Africa and other non-Western countries. First, the psychological effects of prolonged exposure to traumatic experiences such as posttraumatic stress disorder (PTSD) symptoms are expected to be long-term and childhood adversities are known to be associated with adult psychopathology yet most previous studies have used cross-section designs, that restrict causal inference (Shaw 2003; Kessler et al. 2010). Exploring the literature, we have come across only one longitudinal study conducted on the long-term consequences of war in Africa, based in Sierra Leone (Betancourt et al. 2010). Second, existing cross-sectional studies lacked adequate control groups to be able to tease out the specific effects of war experiences on mental health in any given population (Singh and Singh 2010). Third, war-affected populations have often been treated as a single, homogenous group without due regard for differences in age, gender, particular war experiences or other factors liable to result in population heterogeneity (Singh and Singh 2010). Fourth, most previous studies have concentrated on the mental health consequences of war without considering the influence of other contextual variables from the individual, family, local community, and or/wider society that may confer potential risks or protection against long-term mental health consequences of war. Indeed, previous studies showed, somewhat unexpectedly, that for some children who participated in armed groups, it is the experiences they have after returning from captivity that are most troubling and that are likely to keep them on an adverse developmental track (Kohrt, Jordans, Tol, et al. 2008). Fifth, few studies in Africa have considered post-war environment and care in addition to the ecological design that has been adopted for this study. Therefore the studies described in the “Cohort Profile” are of theoretically sound and potentially useful and new findings that will contribute to practice and theory. Finally, most assessment scales used in previous studies were developed and validated in the West, so may be culturally inappropriate for use in Africa (Bracken et al. 1995). In this study, we used mainly locally derived tools.

### What do we know about the risk factors for continued mental health problems in war-affected people?

Survivors of war are at an increased risk of mental ill-health such as post-traumatic stress disorder (PTSD) and conduct problems, but the pathways to, and mechanisms for sustained long-term mental health problems are poorly understood (Betancourt et al. 2010; Wessells 2009; Betancourt 2010; Bolton et al. 2007). For example, a study in Nepal indicated that post-war experiences promote resilience or adverse psychosocial outcomes (Kohrt, Jordans, Tol, et al. 2008) while other studies showed that although the majority of former child soldiers are resilient, contextual community factors such as stigma or discrimination, strongly influence long-term adjustment during re-integration (Betancourt 2010; Wessells 2009). Consequently, potentially exacerbating and mitigating factors that influence the mental health of war-affected youths may include not only their past war experiences but also post-war factors.

The WAYS study will use the Social Ecology Model (Bronfenbrenner 1979; Boothby 2008). This model takes into consideration the complex interplay among individual (e.g. temperament, motivation, coping), family (e.g. family acceptance, relations, functioning, etc.), and community (e.g. stigma/discrimination, community acceptance) contextual factors, thus addressing both the individual and its social ecology (Boothby 2008). In this study, we hope to generate and add to knowledge on post-war contexts that may inform policy and guide interventions to mitigate the long-term mental health problems and war-related traumatic experiences.

### Who is in the sample?

From the districts most affected by the conflict in northern Uganda (Gulu, Nwoya, and Amuru), we recruited 539 (61% male) war-affected youths aged between 18 and 35 years old who met our inclusion criteria of having been abducted and subsequently lived in rebel captivity for at least 6 months and currently accessible via community groups devoted to former abducted people. In Uganda, districts are organised into smaller geographically-defined administrative units known as sub-counties. At every sub-county, formerly abducted youths are members of umbrella organisations which cater for their needs and interests and promote relevant activities. We approached the local authorities at each sub-county and randomly selected the members eligible for the study i.e. those who met the inclusion. In total, 650 participants were invited for baseline assessment and further planned follow-up evaluation and data was collected from 539 of them, representing 83% of those initially invited. Those who did not participate were recorded as either sick, attending to their sick children or were absent for various reasons e.g. some were attending to their farms or funerals.

Eighty six per cent (86%) of the participants were of Acholi ethnic group, 10 percent were Lange ethnic group and the rest were other ethnic groups such as Made, Allure, South Sudanese, etc. At baseline, participants were aged 18 – 25 years 95% of whom had only primary education.

### What is attrition like?

**Figure 3:**
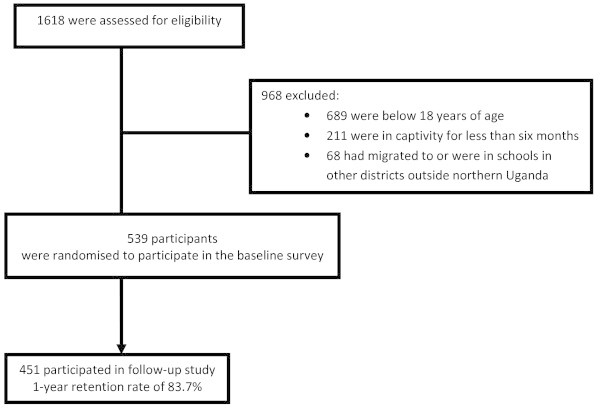
**Flow chart of participants in the WAYS study.**

At follow-up, 451 participants (84%) were interviewed (61% male). Thirty one (6%) could not be located and 53 (10%) were unable to attend for reasons such as sickness, attending to their farms or funerals. To maximise participant retention, we attempted to maintain telephone contacts with those who had mobile phones. Considering the vast region, scattered nature of post-war settlements, and sometimes poor telephone connectivity, the interview at follow-up of 84% was very good (Figure 3). The psychosocial outcomes between responders and non-responders at follow-up are shown in Table 1. In general, the differences between responders and non-responders were minimal and may not have affected the outcome of the study.

**Table 1:** **Descriptive statistics of continuous scores on psychosocial outcomes between responders and non-responders at follow-up**

	Responders					Non-responders			
Psychosocial outcomes	n	Mean	SD	IQR	Differences (t-test)	n	Mean	SD	IQR
Depression/anxiety	451	21.42	10.10	15	ns	88	21.40	11.07	16
Conduct problems	450	2.11	2.94	3	ns	83	1.87	2.95	3
Somatic complaints	449	3.93	2.19	3	ns	87	3.84	2.26	3
Prosocial behaviours	450	10.44	2.18	2	ns	86	10.69	2.34	3
Psychotic symptoms	439	4.04	2.61	4	***p*** **< .05**	88	3.81	2.26	4
Difficulties with daily tasks	450	15.93	11.04	15	***p*** **< .05**	85	17.56	11.07	18
Alcohol and drug abuse	448	1.02	1.86	2	ns	87	1.03	2.24	0

Key: *n* number of male or female participants, *SD* Standard Deviation, *IRQ* interquartile range, *N* total number of participants.

### What variables are measured?

Table 2 shows the specific variables collected at baseline and at follow-up in the WAYS study. The measures included: demographic characteristics (age, sex, education, etc.), pre-abduction experiences, different categories of war experiences, post-war experiences and hardships, contextual factors (individual, family and community), care, and psychosocial outcomes.

**Table 2:** **Variables collected at baseline and follow**-**up in the WAYS study**

	Variables	Wave I	Wave II	Measure used
1	**Demographic characteristics**	√	x	Made for the study
2	**Pre**-**war experiences**	√	x	War Trauma Screening Scale based on the War Trauma Screening Scale (UNICEF, 2010).
3	**War experiences**	√	x	UNICEF Post-war Screening
	Direct personal harm	√	x	
	Witnessing violence	√	x	
	Physical threat to self	√	x	
	Deaths	√	x	
	Harm to loved ones	√	x	
	Material losses	√	x	
	threat to loved ones	√	x	
	Separation	√	x	
	Displacement	√	x	
	Involvement in hostilities	√	x	
	Substance and drug abuse	√	x	
	Sexual abuse/assault	√	x	
4	**Post**-**war experiences**	√	x	
	Post-war hardships	√	x	
	Housing	√	x	
5	**Individual factors**	√	x	Adult Temperament Questionnaire: Evans, D.E., & Rothbart, M.K. (2007). Development of a model for adult temperament. *J of Resin Personality*, 41,868-888.
.	Temperament	√	x	
	Social Skills	√	x	Social Skills Rating System (Gresham & Elliott, 1990)
	General health	√	x	Goldberg D, Williams P (1988) General Health Questionnaire - GHQ, Windsor
	Coping	√	x	Cognitive Emotion Regulation Questionnaire (CERQ) Garnefski N, Kraaij V, Spinhoven, Ph, (2001)
	Motivation	√	x	Myers Achievement Motivation Scale
	Trust	√	x	Made for the study
	Self-esteem	√	x	The Rosenberg Self-Esteem Scale
	Variables	**Wave I**	**Wave II**	**Measure used**
	Posttraumatic growth	√	x	Tesdeschi, RG; Calhoum, LG (1996). Posttraumatic Growth Inventory: Measuring the positive legacy of Trauma. Journal of Traumatic Stress, 9(3); 455-471
	Lethargy	x	√	RRS
	Trauma memory quality	x	√	TMQQ Trauma Memory Quality Questionnaire
	Traumatic reminders	√	x	War Trauma Screening Scale based on the War Trauma Screening Scale (Layne et al. 2010). UNICEF Post-war Screening Survey
6	**Family factors**	√	√	
	Gender-based violence	√	x	UNFPA questionnaire
7	**Psychological**/**psychiatric care**	√	x	Questions specifically made for this study
√	Family functioning	√	x	Survey of Youth Reintegration – Sierra Leone, Version Whitaker et al. 2008, Community and Family acceptance and Community Relations - Teresa S. Betancourt
	Family acceptance	√	x	
	Family relations	√	x	
8	**Community factors**	√	x	
	Discrimination/stigma	√	x	
	Community acceptance			
	Community relations			
9	**Psychosocial outcomes**	√	x	African Youth Psychosocial Assessment Instrument (APAI) Teresa S. Betancourt. Betancourt, TS; Bass, J.;Borisova, I; Neugebauer, R.; Speelman, L.; Onyango, G.; Bolton, P. (2009)
	Depression/anxiety	√	x	
	Conduct problems	√	x	
	Somatic complaints	√	**x**	
	Difficulties with daily tasks	√	x	
	Pro-social behaviours	√	x	
	Psychotic symptoms	√	x	
	Substance and drug abuse	√	x	
	PTSD	√	x	PCL-17

Key: √ = assessed X = not assessed.

### Measures

Participant demographic characteristics. A demographic inventory specifically made for this study was used to collect information on sex, age, duration in captivity, parental status, and number of children. Details of other measures are in Table 2.

### War experiences

To assess individual exposures to different war events, we used items from the War Trauma Screening Scale (UNICEF 2010), initially developed for use in Bosnia and later adapted for use with West African war-affected youth. The questionnaire was adapted by our research team to better capture the context of the war in countries in West Africa (e.g. Sierra Leone, Uganda, DR Congo); for example, items on sexual abuse was added. The adapted instrument contained 52 items capturing a diversity of war-related experiences such as: Personal harm (6 items, e.g. serious injuries), witnessing general war violence (11 items, e.g., massacres or raids on villages), sexual abuse (1 item), and involvement in hostilities (2 items, e.g. did you fight in the army or warring faction?). Other war experiences include: Separation (2 items), Deaths (7 items, e.g. parents, siblings, or extended family members), Material loss (4 items), Physical threat to self (5 items), Harm to loved ones (4 items), Physical threat to relatives or loved ones (4 items), Displacement (5 items), and Drug and substance abuse (1 item). War experiences simply binary coded for occurrence (1) versus absence (0).

### Mental health outcomes

Mental health outcomes were assessed by African Youth Psychosocial Assessment Instrument (APAI), a field-based measure previously developed for use in northern Uganda (Betancourt et al. 2009a; Betancourt et al. 2009b). The measure comprises 40 items (depression/anxiety [18 items], hostility [10 items], pro-social behaviours [5 items], and somatic complaints without medical cause [3 items]). Psychotic symptoms were added [4 items]. Each of these dimensions is represented by a set of questions that inquires about specific behaviours particular to that dimension (e.g., “I do not sleep at night” [Depression], “I fight” [Hostility]. For each question responses were scored on a 1–4 scale, ranging from 0 = never, 1 = rarely, 2 = sometimes, and 3 = always. We computed a subscale score for each participant (e.g., depression/anxiety and hostility. For each question responses were scored on a 1–4 scale, ranging from 0 = never, 1 = rarely, 2 = sometimes, and 3 = always.

### How the measures were administered

Data was collected by university graduates who had been extensively trained in data collection and interviewing skills, and briefed in the study background and detailed interview content. The interviewers visited the participants in their homes or nearby trading centres or community halls to conduct semi-structured face-to-face interviews and also to administer questionnaires covering a wide range of topics. Information sought in the questionnaire include: demographic characteristics (e.g. age, sex, education, marital status, number of children) their experiences before, during, and after war, individual factors (e.g. temperament, coping, etc.), family characteristics (e.g. family functioning), and community characteristics or environmental perceptions, and psychosocial outcomes (common symptoms of depression, anxiety, hostility, pro-social behaviours, psychosis, etc.). The baseline assessment was conducted between June and September 2011 and the follow-up assessment was conducted between July and September 2012.

### Consent

The Institutional Review Board (IRB) of Gulu University approved the WAYS study. Gulu University IRB is an affiliate of Uganda National Council for Science and Technology (UNCST), the overall body that oversees research in Uganda. Subsequently, written informed consent was obtained from all participants in accordance with ethical guidelines and approvals. Written informed consent was obtained from the participants for publication of this report.

### How often will they be followed?

A second data collection phase (first follow-up) took place between July and August 2012. It is anticipated that the sample will be re-assessed in 2014, 2016, and 2018 at which the cohort members will be aged between 25 – 42 years. To limit future attrition, initial data collection has included information on geographical location, next of kin, mobile telephone contacts, and addresses of local leaders, to ease tracking participants in future follow-ups.

### What has it found?

It is evident that both male and female youths experienced the most macabre aspects of the war. Average age at abduction (mean=14.14 years; SD=4.21; min=7; max=34) and 70% were abducted before the age of 15. Duration in captivity (mean = 3.12 years; SD=2.99; min=.05; max=17.5) and exposure to violence was similar in males and females except for sexual abuse (*t* = −15.76, *p*< .001) and involvement in hostilities (*t* = 7.19, *p*< .001). More female than male have spouses who are former abductees or with a military or paramilitary background (*t* = 4.57, *p*< .001). Apparently, there is an indication that marriage may be protective of adverse mental health outcomes and poor functioning as more divorced (Mean=21.5; SD: 10.9) or separated (Mean=17.9; SD= 11.9) youths scored higher on poor functioning than married (Mean=15.1; SD: 9.9) youths. War experiences were significantly associated with difficulties performing daily activities and tasks, more especially in female than in male participants (*t* = −2.62, *p*< .001). Tables 3 and 4 respectively show continuous scores on war experiences and psychosocial outcomes. Women are more at risk of long-term mental health outcomes than men. Male youths scored higher than female youths on alcohol and drug use (*t* = 5.33, *p* < .05) but female participants differed significantly from male participants on depression/anxiety (*t* = −6.03, *p* < .05), somatic complaints (*t* = −5.20, *p* < .05), and difficulties with daily tasks (*t* = −2.62, *p* < .05).

**Table 3:** **Descriptive statistics of mean number of war events experienced**

	Male				Sex differences (t-test)	Female				All			
War experiences	n	Mean	SD	IQR	***p***-value	n	Mean	SD	IQR	N	Mean	SD	IQR
Witnessing violence	329	10.49	.76	1	***p*** **< .05**	209	10.22	1.28	1	538	10.38	1.00	1
Physical threat	328	4.74	.64	0	ns	208	4.71	.63	0	536	4.73	.63	0
Deaths	326	3.90	1.34	2	ns	209	4.06	1.42	2	535	3.96	1.37	2
Harm to loved ones	322	3.88	.39	0	***p*** **< .05**	209	3.73	.71	0	531	3.82	.54	0
Material losses	324	3.94	.31	0	ns	210	3.89	.42	0	534	3.92	.36	0
Threat to loved ones	323	3.04	.96	1	***p*** **< .05**	210	2.90	1.11	2	533	2.99	1.02	2
Displacement	320	3.81	.68	0	***p*** **< .05**	207	3.68	.74	1	527	3.76	.70	1
Involvement in hostilities	324	1.84	.45	0	***p*** **< .05**	208	1.46	.76	1	532	1.69	.62	0
Separation	326	1.94	.29	0	ns	210	1.94	.28	0	536	1.94	.29	0
Total war experiences	301	42.01	3.47	4	ns	202	41.27	5.05	7	503	41.71	4.19	5

Key: *n* number of male or female participants, *SD* Standard Deviation, *IRQ* interquartile range, *N* total number of participants.

**Table 4:** **Descriptive statistics of continuous scores on psychosocial outcomes**

	Male					Female				All			
Psychosocial outcomes	n	Mean	SD	IQR	Sex differences (t-test)	n	Mean	SD	IQR	N	Mean	SD	IQR
Depression/anxiety	323	19.20	9.91	15	***p*** **< .05**	195	24.74	10.49	16	518	21.29	10.47	16
Conduct problems	321	2.00	2.80	3	ns	203	2.26	3.19	3	524	2.10	2.96	3
Somatic complaints	325	3.51	2.19	3	***p*** **< .05**	210	4.50	2.13	3	535	3.90	2.22	4
Prosocial behaviours	326	10.59	2.05	2	ns	208	10.25	2.39	3	534	10.46	2.20	3
Psychotic symptoms	325	3.85	2.70	4	ns	206	4.18	2.52	4	531	3.98	2.64	4
Difficulties with daily tasks	294	14.81	10.41	15	***p*** **< .05**	181	17.50	11.57	18	475	15.84	10.94	17
Alcohol and drug abuse	318	1.37	2.22	2	***p*** **< .05**	200	0.47	1.20	0	518	1.02	1.94	2

Key: *n* number of male or female participants, *SD* Standard Deviation, *IRQ* interquartile range, *N* total number of participants.

A number of manuscripts have been submitted for publication based on the first wave of data collection. In the first article, we studied the impact of different categories of war experiences on depression/anxiety. We found that different categories of war experiences impacted on depression/anxiety differently. For example, among different war events, “witnessing violence”, “deaths”, “threat to loved ones”, and “sexual abuse” were the most noxious predictors of symptoms of depression/anxiety in war-affected youths. Multivariate model yielded unique associations of “witnessing violence” (β = 0.29, SE=0.09, p<.001) and “deaths” (β = 0.14, SE=0.05, p<.001) with symptoms of depression/anxiety irrespective of gender. Sexual abuse (β = 0.32, SE=0.16, p<.001) uniquely predicted symptoms of depression/anxiety for female but not male youths whilst “threat to loved ones” (β = 0.13, SE=0.07, p<.05) uniquely predicted symptoms of depression/anxiety in male but not female youths.

In the second study, we assessed the effects of different war experiences on functioning (participating in daily tasks and activities), the need for mental health services, and barriers to mental health services among veteran child soldiers in northern Uganda. Poor functioning was significantly associated with war experiences and a huge need for and significant barriers to mental health services exist in post-war communities. Poor functioning was significantly predicted by “deaths” (β = .10 (95% confidence interval (CI): .02, 19), “material losses” (β = .11 [CI: .03, .19]), “threat to loved ones” (β = .10 [CI: .02, .20]), and “sexual abuse” (β = .19 [CI: .10, .28]). Only 17% reported having seen a mental health worker despite about 70% acknowledging that they experience emotional and behavioural problems and 73% expressed interest in receiving help. Stigma, fear of family break-up, and lack of health workers and distance to health facilities were identified as barriers to mental health services.

In the third study, we used structural equation modelling to test two competing hypotheses: (1) the “trauma path” in which war experiences directly influences mental health and (2) the “psychosocial path” in which the influence of war experiences influences mental health through post-war hardships. Structural equation modelling indicated that post-war hardships mediated the relations between war experiences and mental ill-health. Significant indirect relationship indicated that war experiences are linked to depression/anxiety through post-war hardships (β = .15 (95% CI: .01, .30)) accounting for 44%, and to hostility (β = .23 (95% CI: .03, .43)) accounting for 89%. Direct relation between war experiences and depression/anxiety attenuated but remained significant. For hostility, the direct relation was no longer significant. Mediation by post-war hardships was larger for hostility than depression/anxiety. Findings support both the “trauma” and “psychosocial” paths. Post-war hardships are key determinants of continued mental ill-health. Policies and interventions to reduce long-term mental ill-health should address both post-war hardships and mental health care.

## Discussion

The scale of atrocities and cruelty in the war in northern Uganda was so grave. Abductees were tortured, forced to kill one another or family members, mutilate villagers, raid their own villages, etc. (Amone-P’Olak 2004; 2005; Amone-P’Olak et al. 2007; Amone-P’Olak 2009). Many of the survivors were made to lick human blood, sit on dead bodies or carry dismembered body parts to imbue courage in them (Amone-P’Olak 2005). The scale of the atrocities and cruelty might explain the enduring noxious effect of “witnessing violence” on symptoms of depression/anxiety. While in captivity, abductees, especially boys, were threatened with death if they tried to escape from rebel captivity or have their villages raided and family members killed if they dared to escape. Indeed this happened to those who tried and succeeded in escaping from rebel captivity. This might explain why “threat to loved ones” significantly predicted symptoms of depression/anxiety. Young girls, especially those assumed not to be sexually active yet, were particularly targeted for abduction by the rebels (Amone-P’Olak 2004; 2005) in the belief that they were free from HIV/AIDS. Very few older women were abducted and taken into captivity. While in captivity, the girls were distributed by senior rebel commanders to loyal, hardworking, and courageous rebel soldiers as “wives”. Indeed, many of the girls returned from captivity with children fathered by rebel soldiers and commanders, usually two or three times older than the girls (Amone-P’Olak 2004; 2005). This may explain the enduring noxious effect of “sexual abuse” on symptoms of depression/anxiety. Results from this sample also showed that although male and female youths experience similar war events, the mental health consequences might be different. For example “involvement in hostilities” remained significantly associated with symptoms of depression/anxiety in female but not in male youths while “threats to loved ones” was significantly associated with symptoms of depression/anxiety in male but not in female youths.

Although the need for mental health services has been identified in previous studies, (Zraly et al. 2011; Tol et al. 2011) no studies have been conducted to assess the need for mental health services yet the majority of war-affected youths continue to function poorly as a result of their war experiences. In this study, poor functioning was independently and uniquely predicted by “deaths”, “material loss”, “threat to loved ones”, and “sexual abuse” categories of war events. The implication of this finding for mental health services is to take into consideration the category of war events experienced in designing mental health services. Sexual violence after war is associated with negative physical, sexual, and reproductive health effects and linked to long-term mental health consequences and child victims are more likely to engage in unsafe sexual practices and be re-victimised later in life (Maniglio 2009; Whitaker et al. 2008). About 70% of the respondents acknowledge that they have a problem and expressed interest in receiving help but only 16.76% reported receiving care from health workers for their mental health problems indicating a huge need for mental health services. Those who received care are apparently better at performing daily tasks and activities. Failure to see mental health or social workers may be due to lack of such workers in the communities or stigma associated with such contacts. Respondents indicated counsellors, social workers, health workers, religious leaders and friends as important sources of help for mental health services. This is an important finding in that these resources for mental health services can easily benefit from training. On the contrary, the finding that local leaders, elders, opinion leaders, and traditional leaders and rituals as sources or agencies for help for mental health problems is significant because their roles had been over-emphasised. Yet, fewer respondents indicated them as sources of help for their emotional and behavioural problems. More studies are required to find out from war-affected youths themselves what they perceive as appropriate sources of care for mental ill-health. Our findings indicate that a small percentage of war-affected youths reported that they had received help from any mental health professional and have better functioning. In resource poor settings where health infrastructure and social services are poor, many other unique factors contribute to resistance and poor access to seeking mental health services. Factors that contribute to poor access to mental health services included fears of stigma or discrimination, break-up of family, lack of means of transport to health facilities often far away or ignorance. Stigma and fear of family break-up disproportionately contributed to inability to access mental health services. This finding has immediate public health implications. Efforts to address the problem of stigma and other barriers to seeking mental health care among war-affected youths should take into consideration outreach, education, and changes in the models of health care delivery, such as increases in the allocation of mental health services at lower primary care levels and in the provision of confidential counselling by means of youth-assistance programs. Ambulatory mental health outreach services are important, especially where mental health care professionals are inadequate. Although there has been anecdotal support for traditional healers or rituals, local leaders, and elders, this study shows less support for such agencies as sources of mental health services. Reducing the perception of stigma and the barriers to care among war-affected youths is a priority for research and for the policymakers, clinicians, and leaders who are involved in providing care to war-affected youths.

The study on the theoretical test of two competing models (the “trauma pathway” and the “psychosocial pathway”) may inform interventions to mitigate the effects of war on war-affected youths. Regarding the “trauma pathway”, the extent of mediation by post-war hardships, especially for depression/anxiety in this study indicates that although interventions that target post-war hardships may provide long-term improvement in depression/anxiety, mental health care remains an important area for consideration (Bolton, Bass, Neugebauer, et al. 2003). In addition, the direct effects of war experiences remained significant for depression/anxiety but not for hostility. The significant direct effect of war experiences on depression/anxiety after adjustment for post-war hardships is consistent with previous findings (Miller & Rasmussen 2010). For example in a study in Mozambique, war-affected boys still experienced intrusive memories related to their war experiences 16 years after they returned to their communities despite showing normal functioning over time (Boothby 2008). This suggests that interventions to diminish the influence of war experiences on some mental ill-health such as depression, should go beyond post-war contexts to include care for war-affected youths in whom the residual effects of war experiences continue to linger on (Yule 2002). Regarding the “psychosocial model” post-war environments provide a logical mechanism through which post-war hardships may influence long-term mental health problems in war-affected youths. War produces or even aggravates extremely stressful situations such as poverty and deprivations, disempowerment, social exclusion, poor housing, gender-based violence as well as community violence, and changes in family configuration and functioning, lack of social support, discrimination/stigma, etc., all of which are linked to mental health problems.

### Scientific plan

Scientific investigations are planned for detailing exposure to war events and their psychosocial consequences in terms of group-based (cluster) analyses, and consideration of pathways to outcomes. Analyses using Structural Equation Model to explain the differences in psychosocial outcomes based on contextual factors measured is currently being undertaken to elucidate the specific pathways from specific war experiences through specific contexts to specific psychosocial outcomes. We also plan to collect data on direct combat and duration of engagement in direct combat to enable us gain insight into extreme trauma. The importance and need for this study has already been published as a letter in a journal (Amone-P’Olak, 2012).

### What are the main strengths and weaknesses?

The current study has strengths. The study is the first to adopt a longitudinal design to study the impact of war experiences on war-affected youths in northern Uganda and the second in sub-saharan Africa after the Sierra Leone study of child soldiers (Betancourt et al. 2010). The large sample size including various sub-populations (community members who were affected by the war but were not abducted, formerly abducted youths married to one another and those married to civilian spouses, and youths who were born in captivity and those born in the communities) nested within the bigger sample of war-affected youths offer possibilities for interesting comparisons yielding information critical to care. Finally, unlike in many previous studies, this cohort uses mainly locally developed and validated scales to assess war experiences, family, and community contextual factors and long-term psychosocial outcomes.

However, this cohort is not without limitations. As is always with other cohort studies, this cohort uses self-report and retrospective assessments, which are limited due to recall bias. Likewise, we were unable to assess mental health before the war, making it difficult to delineate whether the mental health problems are exclusively due to the war as it is not possible to establish temporal relationship between environmental hardships/poverty and negative life events as potential risk factors for mental health problems. Finally, there will be a trade-off between studying wide ranging measures included in the study and detailed investigation.

### Can I get hold of the data? Where can I find out more?

After the initial questions of the study have been answered, The WAYS Study dataset will be made available to be accessed by interested researchers. Researchers will be allowed to use the data after presenting a brief research proposal to be submitted to “The WAYS Study” secretariat and the PI.

### Key messages

-  The WAYS Study comprises a cohort of 539 (61% female) of different sub-populations of war-affected youths aged 18 – 25 randomly sampled from a population of war-affected youths in northern Uganda. The sub-populations of the general population of war-affected population include: those affected by the war but were not abducted, formerly abducted youths, and children (now youths) born in captivity.
-  The youths were recruited from the communities where they currently live together with people who were affected by the war but were not in captivity. Baseline and follow-up data have so far been gathered and more data collections are planned for the future.
-  Our main aim is to assess individual, family and community risk and protective factors for long-term mental health problems to inform interventions aimed at mitigating long-term mental health consequences of prolonged exposure to war-events.
-  There are strong indications that potentially exacerbating and mitigating factors that influence the mental health of war-affected youths include not only their previous war experiences but also post-war contexts. Huge need for mental health services exist in communities emerging from war.

## Conclusion

Longitudinal studies are imperative in unraveling the pathways from traumatic war experiences, post-war contextual risk and protective factors and long-term psychosocial outcomes in war-affected populations. Different categories of war events appear to have different effects on psychosocial outcomes. Post-war environment accounts for long-term psychosocial outcomes. Therefore, potentially exacerbating and mitigating factors that influence the long-term mental health of war-affected youths include not only their previous war experiences but also post-war individual, family, and community contexts. Studying post-war contexts inform interventions to mitigate long-term psychosocial outcomes on war-affected population.
